# The burden of physical activity on type 2 diabetes public healthcare expenditures among adults: a retrospective study

**DOI:** 10.1186/1471-2458-11-275

**Published:** 2011-05-04

**Authors:** Jamile S Codogno, Rômulo A Fernandes, Flávia M Sarti, Ismael F Freitas Júnior, Henrique L Monteiro

**Affiliations:** 1 Department of Physical Education. UNESP Univ Estadual Paulista, Rio Claro, Brazil; 2 Department of Physical Education. Universidade do Oeste Paulista - UNOESTE, Presidente Prudente, Brazil; 3 School of Arts, Sciences and Humanities. Universidade de São Paulo - USP, São Paulo, Brazil; 4 Department of Physical Education. UNESP Univ Estadual Paulista, Presidente Prudente, Brazil

**Keywords:** Diabetes Mellitus, expenditures, physical activity

## Abstract

**Background:**

Determinants of public healthcare expenditures in type 2 diabetics are not well investigated in developing nations and, therefore, it is not clear if higher physical activity decreases healthcare costs. The purpose of this study was to analyze the relationship between physical activity and the expenditures in public healthcare on type 2 diabetes mellitus treatment.

**Methods:**

Cross-sectional study carried out in Brazil. A total of 121 type 2 diabetics attended to in two Basic Healthcare Units were evaluated. Public healthcare expenditures in the last year were estimated using a specific standard table. Also evaluated were: socio-demographic variables; chronological age; exogenous insulin use; smoking habits; fasting glucose test; diabetic neuropathy and anthropometric measures. Habitual physical activity was assessed by questionnaire.

**Results:**

Age (r = 0.20; p = 0.023), body mass index (r = 0.33; p = 0.001) and waist-to-hip ratio (r = 0.20; p = 0.025) were positively related to expenditures on medication for the treatment of diseases other than diabetes. Insulin use was associated with increased expenditures. Higher physical activity was associated with lower expenditure, provided medication for treatment of diseases other than diabetes (OR = 0.19; p = 0.007) and medical consultations (OR = 0.26; p = 0.029).

**Conclusions:**

Type 2 diabetics with higher enrollment in physical activity presented consistently lower healthcare expenditures for the public healthcare system.

## Background

During the last decades, the prevalence of some chronic diseases increased significantly in the worldwide population [1-3]. The burden associated with the outcomes of such diseases generates a substantial impact in public health costs. In particular, Type 2 Diabetes Mellitus (T2DM) may be considered one of the main chronic diseases related to increased healthcare costs, due to the set of co-morbidities derived from its onset.

The economic costs from T2DM complications were estimated to be higher than US$130 billion in the United States during 2002 [4], a situation similar to developing countries. In Latin America, costs due to diabetes were estimated to be US$65 billion during 2000 [5]. Specifically in Brazil, T2DM represents a significant public health cost in hospitalizations [6], especially considering the accelerated process of demographic transition.

Habitual physical activity presents a protective effect against the onset of some diseases, such as overweight and T2DM [7-10]. Diloreto et al. [11] in a longitudinal design carried out with Italian T2DM subjects, identified that increased physical activities during leisure time may result in cost savings in healthcare. Beltoldi et al. [12] also observed a significant negative association between physical activity and the need to use medication.

Despite these results there is still a lack of studies on the association between habitual physical activity and public healthcare costs due to T2DM in developing nations, therefore, there is no evidence that the promotion of physical activity by government could be useful to reduce T2DM treatment costs. Thus, the purpose of this study was to analyze the relation between the level of physical activity and the expenditures in public healthcare with T2DM treatment.

## Methods

### Sample

A cross-sectional study with retrospective design was carried out in the city of Bauru, a large city in the state of São Paulo, Brazil, with approximately 345,000 inhabitants [13]. The study was approved by the Ethics Committee Group from the Universidade Estadual Paulista - UNESP (Rio Claro campus) and all subjects were informed of the aim of the study and their rights not to participate or to abandon the study at any time without constraints. All subjects were asked to sign a standard written consent form (Process number 6898-2008). The sample size was estimated based on the prevalence of physical inactivity among diabetic subjects (47%), using as parameters a 10% error, 80% power and 5% significance, considering an estimated 33,700 diabetic individuals in the population (9.7% prevalence of T2DM in the state of São Paulo). A sample size of 118 diabetic individuals was estimated to be representative and a 10% expected sample loss was included, resulting in the enrollment of 130 T2DM individuals to be included in the study.

In the Brazilian public health system, each Basic Healthcare Unit (BHU) serves the population of a particular region of the city and this is where a variety of health professionals are available (e.g. dentist, general practitioner, gynecologist, obstetrician, pediatrician and psychiatrist) and health services, such as vaccines, medication dispensing and monitoring of patients with arterial hypertension and T2DM are administered [14]. Services are free and focused exclusively on prevention. Emergencies, surgical procedures and complex exams are referred directly to hospitals [14]. BHU services are funded by the State and Municipal Health Secretary. The Municipal Health Secretary granted access to the patients' medical records of two BHU (the city has 17). Potential patients were identified through analysis of the medical records, in which three inclusion criteria were adopted: (i) individuals should have been previously diagnosed with T2DM; (ii) age ≤ 75 years-old, in order to avoid age-related interference in diabetic neuropathy diagnosis [15]; and (iii) individuals would have to present at least one-year complete medical records previous to the interview.

All patients registered at a BHU and who met the inclusion criteria were contacted by phone (n = 186), and 130 were found and agreed to participate. Among the 130 diabetics contacted, nine were excluded due to incomplete data (e.g. fail in body composition assessment and age higher than 75 years) and consequently only 121 individuals were included in the study. Interviews were performed from January to February, 2009. Interviews and all anthropometric measurements were performed by trained researchers.

### Public healthcare expenditures

The period of time considered to assess expenditures for T2DM treatment in public healthcare facilities was one year prior to the interview conducted in the study. Expenditures due to T2DM treatment were estimated including all items registered in the medical records from each T2DM patient in the BHUs. The following data was gathered: laboratory tests performed (performed in private laboratories that were paid by BHU); medical consultations (e.g. dentists, gynecologist, obstetrician, general practitioner and psychiatrist); medication dispensed (separating medication designed for T2DM treatment [e.g. metamorfin, insulin and oral antidiabetic agents] and medication for other diseases [e.g. antihypertensive medicines and hypolipidemic agents]). Hospitalizations and emergency room visits are not services related to BHU and, therefore, were not added to our expenditures.

A specific standard table, including public healthcare reimbursement values, were provided by BHU offices and used in order to compute monetary values of laboratory tests and medical specialist consultations [14]. Invoices obtained from BHUs were used to compute the dosage and the market prices of medication used by T2DM patients. All expenditures were computed in the Brazilian currency (Real) and converted to US dollar using the average value of the dollar against the Brazilian currency in the 12 months of 2008 (US$ 1.83).

Seven categories of healthcare expenditures were created: (i) laboratory tests; (ii) medication ([iii] medication for T2DM treatment and [iv] medication for treatment of other diseases); (v) medical consultations; (vi) nursing consultations; (vii) overall expenditures. For statistical procedures, overall expenditures in T2DM treatment were ranked and stratified into tertiles and the highest tertile of expenditure was adopted as "high healthcare expenditure indicator".

### Diabetic Neuropathy

Diabetic neuropathy was evaluated using 10 g of Semmes-Weinstein monofilament, according to the Brazilian Consensus on Chronic Complications from the Brazilian Society of Diabetes [16]. Touch and pressure perception was tested in the plantar region, in the sensory distribution of the posterior tibial nerve (1^st^., 3^rd^. and 5^th^. metatarsal and 1^st^., 3^rd^. and 5^th^. toes). Each area had its sensitivity tested at least three times, in order to determine the outcome (outcome presence was determined as failing to perceive the monofilament at least one time). Lack of monofilament perception into two or more regions identified the presence of diabetic neuropathy.

### Structured interview and anthropometry

On the same day as the diabetic neuropathy assessment, a structured face-to-face interview and anthropometric measures were carried out in a quiet room at the BHU by a trained researcher. Initially, some data were obtained through interviews and confirmed in the medical records: (i) socio-demographic variables (sex, chronological age [structured as categorical variable: <50 years-old and ≥50 years-old] and skin color [white, black, yellow and other]; (ii) smoking habits (diabetics were categorized as (current smokers [independently of number of cigarettes per day]; former smokers; never smoked); (iii) last fasting glucose test (<99 mg/dL; 100-125.9 mg/dL; ≥126 mg/dL); (iv) exogenous insulin use (either "yes" or "no"); (v) physical activity (described below). In sequence, the following anthropometric measurements were done: body mass index (BMI) and waist-to-hip ratio (WHR) were calculated using measurements of weight, height, waist and hip circumferences done according to Lohman et al. [17]. BMI was obtained dividing weight by squared height (kg/m^2^) and WHR was calculated dividing waist circumference by hip circumference. Presence of obesity was diagnosed when BMI was ≥30 kg/m^2 ^and occurrence of high WHR was diagnosed by gender-adjusted cutoffs (male = 0.95 m and female = 0.80 m) [18].

### Physical Activity Level

Physical activity level was estimated using the questionnaire developed by Baecke et al. [19], which takes into count three physical activity domains: occupational, sports and leisure and mobility. The questionnaire consists of 16 questions and is scored on a 5-point Likert scale, ranging from never to sometimes/very often. Additional questions report the type of sport, number of hours per week and the number of months per year in which this sport was performed [10,19]. The scoring of the questionnaire includes specific scoring criteria for each of the three domains and the sum of the values in all domains represents the total physical activity [10,19]. In order to categorize the physical activity levels registered, the formula proposed by Baecke et al. [19] was used. The sample was divided into quartiles according to total physical activity score provided by the instrument: 1^st^. Quartile ([mean score = 4.73 and 95%CI = 4.51 - 4.94] Sedentary, n = 31), 2^nd^. and 3^rd^. Quartile ([mean score = 6.21 and 95%CI = 6.06 - 6.36] Moderately Active, n = 58); and 4^th^. Quartile ([mean score = 8.94 and 95%CI = 8.29 - 9.58] Active, n = 32).

### Statistical procedures

Numerical variables were presented as mean and standard-deviation (SD). Student t test compared mean values between men and women. Pearson correlation analyzed the relationship between physical activity and healthcare expenditures. Categorical data were expressed as rates. Chi-square tests were applied to detect the existence of significant associations among categorical variables (Yates' correction was used in 2 × 2 contingence tables). A multivariate model was structured using binary logistic regression (expressed as odds ratios [OR] and 95% confidence intervals [95%CI]), inserting potential confounding variables significant in univariate chi-square (all categorized) to analyze the association between physical activity and healthcare expenditures. All statistical procedures were performed in the Statistical Package for the Social Sciences (SPSS), version 13.0, and the significance (p) was set at 5%.

## Results

The mean age was 60.1 ± 8.9 years, ranging from 32.7 to 75.0 years. The mean BMI and fasting glucose were 30.6 ± 6.5 kg/m^2 ^and 164.9 ± 72.1 mg/dl, respectively. The number of medical consultations per year was 1.83, ranging from 0 to a maximum of 6. Table 1 shows the general characteristics of the sample grouped, according to gender. Mean values of public health expenditures and physical activity score were similar in men and women (p > 5%), but women presented higher expenditures on medication for other diseases (p = 0.006). The sample included mainly white individuals, presenting poor glycemic control and high percentage of subjects with increased adiposity. Women presented higher prevalence of overweight and obesity (p = 0.008) and elevated WHR (p = 0.003). Physical activity level was not associated with gender (p = 0.586).

**Table 1:** Socio-demographic, anthropometric and health characteristics in a sample of type 2 diabetic individuals, according to gender (Brazil, 2009).

Variables	Overall (n = 121)	Male (n = 49)	Female (n = 72)	P
	Mean ± SD	Mean ± SD	Mean ± SD	
Age in years	60.1 ± 8.9	59.8 ± 9.9	60.2 ± 8.3	0.802
BMI (kg/m^2^)	30.6 ± 6.5	28.2 ± 4.9	32.3 ± 7.1	0.001
Physical activity score	6.5 ± 1.8	6.6 ± 1.8	6.4 ± 1.8	0.663
Medical consultations per year	1.83 ± 1.4	1.86 ± 1.5	1.82 ± 1.3	0.884
Laboratory tests (US$)	14.5 ± 1.3	14.2 ± 2.1	14.7 ± 1.7	0.872
Medication (US$)	89.1 ± 10.8	98.8 ± 17.4	82.1 ± 13.8	0.461
T2DM medication (US$)	81.9 ± 10.9	93.7 ± 17.4	73.9 ± 13.9	0.376
Other medication (US$)	7.1 ± 0.6	5.1 ± 0.7	8.5 ± 0.9	0.006
Medical consultation (US$)	7.8 ± 0.4	8.1 ± 0.8	7.9 ± 0.5	0.859
Nursing consultation (US$)	37.4 ± 1.2	39.5 ± 1.3	35.9 ± 1.9	0.128
Overall expenditures (US$)	148.9 ± 11.7	160.5 ± 17.7	140.5 ± 14.1	0.481
Skin color (%)				0.361
White	71.9	73.5	70.8	
Black	18.2	10.2	23.6	
Yellow	6.6	10.2	4.2	
Other	3.3	6.1	1.4	
Fasting glucose (%)				0.238
<99 mg/dl	9.1	10.2	8.3	
100-125 mg/dl	22.3	28.6	18.1	
≥126 mg/dl	68.6	61.2	73.6	
WHR (%)				0.003
Normal	13.2	24.5	5.5	
High	86.8	75.5	94.4	
BMI (%)				0.008
<25 kg/m^2^	18.2	24.5	13.9	
25-29.9 kg/m^2^	35.5	44.9	29.2	
≥30 kg/m^2^	46.3	30.6	56.9	
Smoking habit (%)	11.6	16.3	8.3	0.289
Exogenous insulin use (%)	36.4	42.9	31.9	0.302
Diabetic neuropathy (%)	26.4	32.7	22.2	0.286
Physically active (%)	26.4%	24.5	27.8	0.586

SD = standard-deviation; BMI = body mass index; WHR = waist-to-hip ratio; T2DM = type 2 diabetes mellitus

In our sample, the total expenditures were approximately US$ 18,500 and medication for T2DM represented 58.4% of these overall expenditures (medication for other diseases 4.7% and medication for T2DM treatment 53.7%). Nursing consultations, medical consultations and laboratory tests represented 24.5%, 7.6% and 9.5% of the overall cost, respectively.

Diabetic individuals aged over 50 years presented higher expenditures on medication for the treatment of diseases other than T2DM (p = 0.041). Smoke habit was not associated with any expenditure indicator (Table 2). As could be expected, insulin use was associated with increased expenditures in general medication (p = 0.001) and specifically for T2DM treatment (p = 0.001). Additionally, insulin use was also associated with higher expenditures in medical consultations (p = 0.005), nursing consultations (p = 0.001) and overall public healthcare expenditures (p = 0.001).

**Table 2:** Higher public healthcare expenditures prevalence in a sample of type 2 diabetic subjects with different socio-demographic and health characteristics, according to expenditure categories (Brazil, 2009).

Independent variables		Expenditure category: higher tertile (n [%])						
		
		i	ii	iii	iv	v	vi	vii
		Laboratory tests	Medication	T2DM medication	Other medication	Medical consultation	Nursing consultation	Overall expenditure
Age	<50 years-old	11 (37.9)	12 (41.2)	12 (41.4)	5 (17.2)	13 (44.8)	9 (31)	12 (41.4)
	≥50 years-old	29 (31.5)	28 (30.4)	28 (30.4)	37 (40.2)*	27 (29.3)	40 (43.5)	28 (30.4)
Smoking habit	No	35 (32.7)	36 (33.6)	36 (33.6)	40 (37.4)	34 (31.8)	42 (39,3)	36 (33.6)
	Yes	5 (35.7)	4 (28.6)	4 (28.4)	2 (14.3)	6 (42.9)	7 (50)	4 (28.6)
Exogenous insulin use	No	24 (31.2)	0 (0)	0 (0)	27 (35.1)	18 (23.4)	20 (26)	0 (0)
	Yes	16 (36.4)	40 (90.9)**	40 (90.9)**	15 (34.1)	22 (50)**	29 (65.9)**	40 (90.9)**
BMI	<29.9 kg/m^2^	19 (29.2)	24 (36.9)	24 (36.9)	22 (33.8)	21 (32.3)	30 (46.2)	24 (36.9)
	≥30 kg/m^2^	21 (37.5)	16 (28.6)	16 (28.6)	20 (35.7)	19 (33.9)	19 (33.9)	16 (28.6)
WHR	Normal	5 (31.3)	5 (31.3)	5 (31.3)	2 (12.5)	6 (37.5)	5 (31.3)	5 (31.3)
	High	35 (33.3)	35 (33.3)	35 (33.3)	40 (38.1)*	34 (32.4)	44 (41.9)	35 (33.3)
Diabetic Neuropathy	No	30 (33.7)	29 (32.6)	29 (32.6)	33 (37.1)	32 (36)	37 (41.6)	29 (32.6)
	Yes	10 (31.3)	11 (34.4)	11 (34.4)	9 (28.1)	8 (25)	12 (37.5)	11 (34.4)
	Sedentary	13 (41.9)	12 (38.7)	12 (38.7)	16 (51.6)	14 (45.2)	13 (41.9)	12 (38.7)
Physical activity	M. Active	20 (34.5)	18 (31)	18 (31)	20 (34.5)	20 (34.5)	24 (41.4)	18 (31)
	Active	7 (21.9)	10 (31.3)	10 (31.3)	6 (18.8)^§^	6 (18.8)^§^	12 (37.5)	10 (31.3)

§ = p < 0.05 compared to Sedentary; * = chi-square test with p < 0.05; ** = chi-square test with p < 0.01; M. Active = moderately active; T2DM = type 2 diabetes mellitus; BMI = body mass index; WHR = waist-to-hip ratio.

WHR was positively associated with expenditures on medication for diseases other than T2DM (p = 0.045). Diabetic neuropathy was not associated with increased expenditures in healthcare (Table 2). There was an association between physical inactivity and public expenditures in healthcare for medication to treat diseases other than diabetes (p = 0.006) and medical consultations (p = 0.026). Sedentary and moderately active diabetics presented similar results (p > 5% in all comparisons).

The logistic regression results (Table 3) show that physically active diabetic individuals were 81% less likely to be in the highest tertile of expenditures due to treatment of other diseases (p = 0.007) and 74% less likely to be in the highest tertile of expenditures on medical consultations (p = 0.029) when compared to sedentary diabetic individuals. Expenditures on laboratory tests presented only a marginally significant association (decreased odds of 62%; p = 0.092).

**Table 3:** Logistic regression results to determinate association with public healthcare expenditures in type 2 diabetics (Brazil, 2009).

Independent variable		Expenditure category (higher tertile)						
		i	ii	iii	iv	v	vi	vii
		
		Laboratory tests	Medication	T2DM medication	Other medication	Medical consultation	Nursing consultation	Overall expenditure
		OR_adj1 _(95%CI)	OR_adj2 _(95%CI)	OR_adj1 _(95%CI)*	OR_adj3 _(95%CI)	OR_adj2 _(95%CI)	OR_adj2 _(95%CI)	OR_adj2 _(95%CI)
PAL	Sedentary	1.00	1.00	1.00	1.00	1.00	1.00	1.00
	M. Active	0.72 (0.29-1.78)	0.70 (0.28-1.77)	0.71 (0.28-1.77)	0.40 (0.15-1.05)	0.33 (0.25-1.61)	1.02 (0.39-2.67)	0.70 (0.28-1.77)
	Active	0.38 (0.12-1.16)	0.69 (0.25-2.03)	0.72 (0.25-2.03)	**0.19 (0.06-0.64)**	**0.26 (0.08-0.87)**	0.87 (0.29-2.62)	0.69 (0.25-2.03)

* = insulin use was significantly associated with T2DM in chi-square test, however, it was not inserted in logistic regression as adjust; OR = odds ratios; OR_adj1 _= odds ratio adjusted by gender and skin color; OR_adj2 _= odds ratio adjusted by gender, skin color and insulin use; OR_adj3 _= odds ratio adjusted by gender, skin color, age and waist-to-hip ratio; 95%CI = 95% confidence interval; T2DM = type 2 diabetes mellitus; PAL = physical activity level; M. Active = moderately active

Age (r = 0.20; p = 0.023), BMI (r = 0.33; p = 0.001) and WHR (r = 0.20; p = 0.025) also were related to medication for other diseases. Physical activity score was negatively related to expenditures on medical consultations (r = - 0.23; p = 0.011) and medication for other diseases (r = - 0.23; p = 0.010).

## Discussion

Older diabetic individuals presented higher expenditures on medication for the treatment of diseases other than T2DM. Similarly, Plotnikoff et al. [20] identified that age was positively related to healthcare utilization in Canadian persons with both types of diabetes. In fact, ageing is frequently pointed out as an independent risk factor for development of several chronic diseases, including T2DM [1,3,8,21]. Additionally, the period of disease onset during the lifespan may be a determinant of the magnitude in healthcare expenditures, due to the co-morbidities associated to prolonged diabetes [22,23].

Regular use of exogenous insulin increased healthcare expenditures significantly with T2DM treatment among all patients, an expected result, since exogenous insulin is one of the most expensive medications financed by the Brazilian public health system. Higher expenditures on medical consultations and nursing consultations among exogenous insulin users, however, were relatively unexpected results. Nevertheless, the major part of the difference identified was related to consultations for medication dispensing, that is, insulin-dependent patients would require more frequent consultations than non-insulin-dependent patients, given that insulin demand special caution in storage and transportation. Furthermore, exogenous insulin is a pharmacological tool used by diabetic subjects with poor glycemic control and, in turn, with more complications of the disease. Khattab et al. [23] evidenced that diabetics with combined use of insulin and oral antidiabetic agents have lower glycemic control than diabetics with only oral antidiabetic agents. Our hypothesis is strengthened, because 69% of the diabetics presented low effectiveness in glycemic control. Thus, the presented results indicated that there is high proportion of diabetic patients with poor glycemic control and this event strongly increases the healthcare costs.

In obese persons, evidence indicates that adipose tissue (mainly visceral) releases a variety of adipokines associated with insulin resistance, dyslipidemia and arterial hypertension [24], suggesting that obesity has central role in the genesis of T2DM. Worldwide being overweight and obese have been associated with increased healthcare expenditures among adult populations [6,25]. The results presented in this paper reinforce evidence previously published, since higher BMI and WHR were significantly related to higher expenditures on medication for chronic diseases other than diabetes. Moreover, our findings call attention to the high occurrence of general and central obesity among individuals with T2DM.

Diabetic neuropathy was not related to increased costs, disagreeing with previous data, in which the presence of diabetic neuropathy significantly increased healthcare costs [26]. Even with the presence of foot ulceration in some patients (n = 8), none of these had either previous diagnosis of diabetic neuropathy or received medication for the treatment of neuropathy (e.g. pain medication). This lack of preventive care related to diabetic neuropathy would justify the absence of differences and constitutes a concern due to the increased risk of ulceration.

Diabetic individuals engaged in physical exercise for at least 30 minutes per day present better glycemic control and lower medication use than sedentary ones [12,23] and this relationship appears stronger in T2DM than type 1 [20]. The present study observed the same patterns in the association between habitual physical activity and expenditures on medication for treatment of diseases other than diabetes (e.g. antihypertensive and hypolipidemic agents). There is a well-documented beneficial effect of physical activity in metabolic and cardiovascular outcomes. Regular participation in physical activities acts on different biologic pathways increasing nitric oxide release, clearing of fat free acids and decreasing in oxidative stress, physiological processes associated with a decrease in the risk for arterial hypertension, dyslipidemia and T2DM [27-29]. Among type 2 diabetic individuals, higher physical fitness is inversely related to blood pressure, body fatness, uric acid and total cholesterol [30]. Therefore it is possible that such multiple beneficial effects from habitual physical activities may be the reason for the decrease identified in public healthcare expenditures due to medication and laboratory tests.

In fact, physically active diabetic individuals are less susceptible to disease complications, resulting in lower expenditures on general medical consultations. On the other hand, the lower number of general medical consultations could also be associated to a better self-perception of health, since physical activity has also been significantly associated with a better quality of self-perception of health [31].

It is noteworthy that our study only calculated expenditures related to preventive medicine, while expenditures generated by complications of the T2DM were not (hospitalization and emergency room). Our results suggests that the impact of large preventive actions targeting diabetic patients attended by public health care system can have relevant contribution in the reducing both the occurrence and the costs of these secondary outcomes. Thus, it will also be necessary that health professionals orientate diabetic patients in leading a healthy lifestyle by means of physical activity and other healthy behaviors.

Limitations must be recognized. First, the cross-sectional design does not offer support for causality statements in the relationship between healthcare expenditures and physical activity level. Second, metabolic equivalents, steps, counts and time of activity are commonly used to describe physical activity and, therefore, there are guidelines based on these variables. On the other hand, the Baecke questionnaire provides a specific score without previously proposed cutoff, limiting the generalization of the results. Third, the burden of both hospitalizations and other diabetic complications/co-morbidities in overall expenditures had not been calculated and, therefore, should also be identified as both a limitation and a possible focus for future studies analyzing the relationship between physical activity and healthcare expenditures among diabetic patients.

## Conclusions

In summary, the results indicate that type 2 diabetic patients attending public healthcare units with higher engagement in physical activity presented consistently lower healthcare expenditures for the public healthcare system. The decrease observed in expenditures to treat the diabetic patients was mainly due to the reduction in medicines used to treat diseases other than T2DM and medical consultations, which represent approximately 12% of the overall cost related to preventive services. Therefore, habitual physical activity promotion could be an important strategy for healthcare systems to decrease costs associated with the treatment of diabetes and its co-morbidities.

## List of abbreviations

95%CI: 95% confidence interval; BHU: basic healthcare unit; BMI: body mass index; OR: odds ratio; SD: standard-deviation; T2DM: type 2 diabetes mellitus; WHR: waist-to-hip ratio.

## Competing interests

The authors declare that they have no competing interests.

## Authors' contributions

JSC: (1) the conception and design of the study, acquisition of data and analysis and interpretation of data, (2) drafting the article and revising it critically for important intellectual content, (3) final approval of the version to be submitted. RAF: (1) Acquisition of data and analysis and interpretation of data, (2) drafting the article and revising it critically for important intellectual content, (3) final approval of the version to be submitted. FMS: (1) revising it critically for important intellectual content, (2) final approval of the version to be submitted. IFFJ: (1) revising it critically for important intellectual content, (2) final approval of the version to be submitted. HLM: (1) the conception and design of the study (2) revising it critically for important intellectual content, (3) final approval of the version to be submitted.

## Pre-publication history

The pre-publication history for this paper can be accessed here:

http://www.biomedcentral.com/1471-2458/11/275/prepub
